# A novel ‘practical body image’ therapy for adolescent inpatients with anorexia nervosa: a randomised controlled trial

**DOI:** 10.1007/s40519-020-00997-2

**Published:** 2020-09-19

**Authors:** Hannah Biney, Sarah Astbury, Amanda Haines, Jessica Grant, Nicola Malone, Matt Hutt, Rachel Matthews, John F. Morgan, Sarah White, J. Hubert Lacey

**Affiliations:** 1 Newbridge House, Birmingham, UK; 2 St. George’s, University of London, London, UK

**Keywords:** Body image, CBT, Mirror exposure, Adolescents, Anorexia Nervosa

## Abstract

**Purpose:**

To determine the potential effectiveness of a novel 10-week manualised Practical Body Image therapy (PBI) with mirror exposure (ME), when used as an adjuvant to an intensive treatment package (TAU) in adolescent inpatients with Anorexia Nervosa (AN). To evaluate the effectiveness of ME in an adolescent population.

**Methods:**

Using a randomised control design, 40 girls aged 11–17 years with AN were assigned to PBI with TAU (*n *= 20) and TAU alone (*n *= 20). Both groups completed self-report measures of body image at week 1 and week 10 of the study to measure the potential effectiveness of PBI. The PBI group completed measures at week 7 to evaluate the ME component.

**Results:**

31 participants completed the study; 16 TAU, 15 PBI. PBI participants had greater improvement in all outcomes than TAU participants. Medium effect sizes were seen for self-reported weight concern, body image avoidance in terms of clothing and body image anxiety. ME produced effect sizes in self-reported body image avoidance in terms of clothing and grooming that were greater than 0.40, *n *= 14.

**Conclusion:**

The findings demonstrate that PBI supports an intensive inpatient treatment package and addresses elements of negative body image. PBI was beneficial for addressing body image dissatisfaction with improvements in weight concerns, body image avoidance and physical appearance trait anxiety following the ME component. The magnitude of the effect sizes is comparable to previous studies. Positive qualitative feedback indicated the intervention was acceptable to users. PBI is a promising new adjuvant treatment for AN.

**EMB Rating:**

Level I: randomized controlled trial.

## Introduction and aims

A core diagnostic feature of Anorexia Nervosa (AN) is body dissatisfaction, defined as ‘a disturbance in the way in which body weight or shape is experienced with undue influence of body weight or shape on self-evaluation or persistent lack of recognition of the seriousness of current low body weight’ [1]. Body dissatisfaction often persists following re-feeding even if other symptoms are diminished [2–5]. Body dissatisfaction has been linked to increased risk of dietary restriction, and is one of the most consistent and robust risk and maintenance factors of eating pathology in adolescents [6]. It has been found to be a reliable predictor of relapse of AN [7–9]. This, therefore, raises the question of how body dissatisfaction can be tackled effectively to provide a holistic treatment for AN to ensure sustained, long-term recovery for patients.

Body image is a complex concept incorporating cognitive, affective and behavioural components. Dysfunction in one or more components can lead to over-estimation of weight and shape [10, 11], negative thoughts and feelings towards the body [12, 13], as well as avoidance and checking behaviours [14, 15]. Therefore, a multi-faceted treatment which specifically targets negative body image and increases body acceptance at a healthy weight could be the best approach.

There is evidence to suggest that cognitive behaviour therapy (CBT)-based group interventions are effective at reducing body dissatisfaction in people with AN. Morgan, Lazarova, Schelhase and Saeidi [16] found that a ten-session mindfulness-based CBT group, including mirror exposure (ME, see below) reduced body checking, body avoidance and anxiety as well as shape and weight concerns in adult inpatients with AN.

In support of the above, Legenbauer, Schutt-Stromel, Hiller and Vocks [17] found a significant reduction in dysfunctional cognitions in an adult outpatient population who received a body image group therapy. Similarly, Bhatnagar, Wisniewski, Solomon and Heinberg [18] found significantly less body image dissatisfaction following a CBT group intervention. The groups targeted attitudinal and behavioural components of body image dissatisfaction for both patients receiving intensive outpatient treatment as well as those receiving lower levels of care, compared to wait list controls.

A ten-session programme was found to improve cognitive-affective and behavioural components of negative body image in adult women with eating disorders, however, no improvements were found for the perceptual component of body image dissatisfaction [19]. Psycho-educational groups based on CBT principles have also been evidenced to reduce body image dissatisfaction in adults [20] and low-weight adolescents [19] with AN. There is also evidence to suggest the effectiveness of CBT-based individual interventions for treating body dissatisfaction in non-clinical populations [22, 23].

Recently, there is a greater focus on body image in manualised treatment approaches such as Cognitive Behaviour Therapy–Enhanced (CBT-E) [24] and Waller et al. [25] CBT manual. Support for this is shown in Calugi and Dalle Grave’s [26] research which found that the completion of CBT-E was associated with a significant improvement in body image concerns and eating disorder psychopathology in adolescents with AN.

Body dissatisfaction is associated with high levels of anxiety when the body is self-examined leading to avoidance of the body or excessive body checking. ME is a method of reducing anxiety, whereby individuals with AN, approaching a healthy weight, are asked to stand in front of a full-length mirror wearing minimal outdoor clothing to view their body shape and size i.e. underwear, a swimsuit or tight fitted clothing for set periods of time. During this time, they are asked to look at parts of their body without avoiding areas of the body which cause concern or anxiety. The aim is to habituate to the fear caused by seeing themselves at a healthy weight body.

The first clinical support for ME came from work led by the last author [27]. This study, conducted in an in-patient setting, compared two group body image interventions for AN. Only the treatment involving ME, used in inpatients nearing a healthy weight, produced significant improvements in body dissatisfaction, reducing body anxiety and avoidance behaviours. This same treatment also led to significant improvements in body image in weight restored patients [16].

Moreno-Domínguez, Rodríguez-Ruiz, Fernández-Santaella, Jansen, and Tuschen-Caffier [28] compared the effectiveness of ME and guided ME (where participants are guided to describe their body in a non-judgmental manner) in non-eating disordered women with body dissatisfaction. Both interventions were found to be effective, however, ME alone was found to be more effective than guided exposure in reducing body dissatisfaction.

ME is a central component of body image interventions and has been found to reduce body image dissatisfaction and body avoidance behaviours in women with and without eating disordered symptoms [29]. In a systematic review of exposure therapy interventions of various forms, ME, completed alone or in the context of CBT, was effective in decreasing body dissatisfaction [30].

In summary, though the evidence base is not large, CBT-based therapies and ME interventions seem to improve body dissatisfaction in both adult women with AN and non-clinical adult samples [31]. This is particularly so when subjects with AN approach their healthy weight. However, to the authors’ knowledge, research into the effectiveness of individual CBT-based interventions for adolescent girls with AN is limited. Recognising a gap in the clinical literature, the authors developed a manualised, individual body image therapy for young children and adolescents with AN known as Practical Body Image (PBI).

The aim of this study was to evaluate the potential effectiveness of PBI which uses CBT approaches and ME to improve body image in adolescents with a diagnosis of AN, when used as an adjuvant to an array of therapies in an inpatient unit. The literature suggested it would be best to conduct the treatment when the patients were nearing a healthy weight to avoid habituation at a lower weight [16]. It also aimed to investigate the therapeutic benefit of ME, which is a specific component of PBI as an intervention for reducing body image anxiety. Qualitative feedback from participants in the intervention group was also sought to evaluate the acceptability and experience of the intervention.

## Methods

### Participants

Participants were child and adolescent girls with a primary diagnosis of AN currently receiving inpatient treatment for their eating disorder at Newbridge House. Diagnosis was established at admission using DSM-5 criteria [1]. Participants were recruited for the study just before reaching a median BMI of 85%, and those meeting inclusion criteria began active participation in the study after reaching a median BMI of 90%. This weight criterion ensured participants had sufficient time to complete the study before discharge. It also meant the subjects reached their minimum healthy weight shortly after starting PBI treatment thereby avoiding habituation at a lower weight for those in the treatment group [16].

Inclusion criteria included a primary diagnosis of AN, aged between 11 and 18 and currently receiving inpatient treatment. Exclusion criteria included previously completing PBI treatment during the development stage, a primary diagnosis other than AN, severe learning difficulty, active psychosis or detainment under the mental health act. Inclusion and exclusion criteria were assessed using clinical documentation or through discussions with the multi-disciplinary team.

A sample size of 40 was considered acceptable to address the objectives of this pilot study. By randomising 20 participants into each treatment arm, differences between groups with respect to self-reported body image anxiety could be estimated with 95% confidence intervals.

Eighty child and adolescent girls with AN were assessed for eligibility to take part in the study, of which 40 consented, meaning that there was a refusal rate of 50% for participation in the study. Prior written informed consent was gained from all patients and their parents. The consenting procedure and forms were agreed and authorised by the West Midlands-Black Country NHS Ethics Committee. The study was reviewed by the Newbridge Research and Ethics Committee. IRAS project ID: 189223.

### Therapy

PBI is a novel, individual body image therapy which has been designed and developed at Newbridge House over several years before being manualised into a 10-week programme. Development of the programme involved drawing from a range of evidence-based techniques for treating body dissatisfaction, collating these into an intensive programme and making revisions based on service user feedback following a period of initial testing.

PBI follows a CBT approach and consists of 14 sessions aimed at identifying and challenging various aspects of negative body image including; body misperception, body avoidance, negative beliefs and body related anxiety. The final six sessions of the programme involve the completion of ME, with the aim of reducing anxiety and increasing acceptance of the body at a healthy weight. See Table 1 for details of the programme structure.

**Table 1:** Programme structure

Week	Session number (session length)	Session outline
1	0: Introduction to PBI (30 min)	[≥ 90% of healthy weight] Introduce programme and complete time 1 Body Image questionnaires
1	1: My body image (60 min)	Using a timeline of body image events to explore how negative body image developed, identify negative beliefs about their body and explore perception and ideal body shape using photographs of different body shapes and sizes
2	2: Body Perception (60 min)	Investigate perception of their body weight and size using a 2D drawing task of their body outline and 3D drawing task of 2-3 body parts
3	3. Body Avoidance Planning and Testing Beliefs (60 min)	Identify body avoidance and use an anxiety ladder to create a hierarchy of avoided clothes. Plan for avoidance outing and survey questions
4	4: Body Avoidance Outing 1 (120 min)	Outing to try on avoided clothes and explore clothes sizes
5	5. Body Avoidance Outing 2 (120 min)	[≥ 95% of healthy weight] Outing to try on avoided clothes and explore clothes sizes and to take photographs at healthy weight for survey
6	6. Review and Reflection (60 min)	Reflect on photographs at different weights, plan how to dispose of ill-fitting clothes and set goals
7	7. Introduction to Mirror Use (60 min)	Survey feedback. Explore healthy mirror use and introduce the rationale for mirror exposure. Complete time 2 Body Image questionnaires
7	8. Mirror Exposure 1 (60 min)	30 min of mirror exposure, reflection and goal setting
8	9. Mirror Exposure 2 (60 min)	30 min of mirror exposure, reflection and goal setting
8	10. Mirror Exposure 3 (60 min)	30 min of mirror exposure, reflection and goal setting
9	11. Mirror Exposure 4 (60 min)	30 min of mirror exposure, reflection and goal setting
9	12. Mirror Exposure 5 (60 min)	30 min of mirror exposure, reflection and goal setting
10	13. Mirror Exposure 6 (60 min)	30 min of mirror exposure, reflection and goal setting
10	14. Ending Session (30 min)	Review progress including negative beliefs about their body image from session 1 and any remaining goals. Complete time 3 Body Image questionnaires and evaluation form

Photographs are taken of the patient at different weights (at 85% and 95% of a healthy weight) wearing fitted clothing, for example leggings and a vest top. Three full-length photographs are taken; facing forwards, facing right, facing back. These photographs are used in session seven.

For the ME element of the programme, the patient is required to stand in front of the mirror for 30 min in fitted clothing, for example leggings and a vest top. They are encouraged to wear the same clothes for each session. Throughout the exposure, they are asked to rate their anxiety every 5 min on a scale of 0–10, whereby 0 is no anxiety and 10 is extreme anxiety. This anxiety rating is recorded on a graph by the therapist. Following the exposure, the patient is asked to reflect on their anxiety ratings and consider what was happening when anxiety reduced, increased or remained the same, i.e. were they scrutinizing body parts or avoiding the mirror.

It is also expected that work is completed outside of sessions and that techniques are put into practice, including the completion of weekly body avoidance challenges.

### Treatment as usual

TAU refers to the standard inpatient treatment programme at Newbridge House which includes: individual and group support with Occupational Therapists, Dieticians, Nurses, Psychologists and Psychiatrists and a leisure programme. Some of these treatments were practical dealing with meals or food preparation, others were psychological addressing body image, self-esteem or family therapy. Other groups were psycho-educational. Medication is rarely used and always briefly, details have not been included in this research project. All treatments took place around the in-house school teaching programme which maintained the children’s education. Details of all these activities can be found on the Newbridge House website.

### Measures

*The Eating Disorder Examination Questionnaire* (EDE-Q) [32] is a 28 item self-report measure developed from the Eating Disorder Examination (EDE) interview-based assessment tool [33], which assesses the core psychopathology of eating disorders. It includes four subscales: restraint, eating concern, shape concern and weight concern. The EDE-Q asks individuals to answer questions based on the previous 4 weeks on a six-point scale. A scoring system has been utilised whereby higher scores on the EDE-Q indicate greater eating disorder psychopathology. For the purpose of this study, only the shape and weight concern subscales were used as these were most relevant to body image and Cronbach’s alpha value were reported as 0.92 and 0.89 for shape and weight concern subscales respectively over a two-week period when tested in undergraduate females. Stability of 0.94 and 0.92 [34].

*The Physical Appearance State and Trait Anxiety Scale*—*Trait Version* (PASTAS) [35] is a 16-item self-report questionnaire which asks people to rate how often they worry about specific body parts in general as a measure of trait body image anxiety using a 5-point scale. A scoring system has been utilised whereby higher scores on the PASTAS indicate greater body image anxiety. The PASTAS has good internal consistency (0.88) and test–retest reliability (0.87).

*The Body Image Avoidance Questionnaire* (BIAQ) [36] is a 19-item self-report questionnaire which measures the different behavioural tendencies indicative of body image avoidance using a 6-point scale. It includes four subscales: clothing avoidance, avoidance of grooming and weight, eating restraint and avoidance of social activities. A scoring system has been utilised whereby higher scores on the BIAQ indicate greater body image avoidance. The BIAQ has good internal consistency (0.89) and test–retest reliability (0.87).

*The Body Image Acceptance and Action* Questionnaire (BIAAQ) [37] is a 12-item self-report measure which assesses body image flexibility using a 7-point scale. A scoring system has been utilised whereby a higher score on the BIAAQ indicate poorer body image flexibility. The BIAAQ has good internal consistency (0.93) and test–retest reliability (0.80).

#### *The feedback form*

Qualitative feedback was collected following completion of the programme, the feedback form asked the young people to rate each session out of 10 for how helpful they had found it, with 1 being not at all helpful and 10 being extremely helpful. It also included open-ended questions asking the young people “what did they find most helpful?”, “was there anything about the sessions that was unhelpful?” and “how would you improve the programme?”.

### Procedure

Patients approaching a median BMI of 85% were assessed by the Research Team at Newbridge House for suitability for the study using the framework of inclusion and exclusion criteria. The Research Team also work clinically as Assistant Psychologists. Those meeting inclusion criteria were then approached by an Assistant Psychologist who introduced the research, provided an information sheet about the study and sought informed consent for participation. Both parents and patients were given 7 days to consent to the research and were informed that they could withdraw at any time.

Patients for whom we received appropriate consent were then randomly allocated to a treatment or control group using an Excel formula, where treatment involved receiving PBI as well as TAU and control involved TAU only. Those allocated to treatment then had full-length photographs taken in a fitted vest top and leggings from the front, side and back when they reached 85% median BMI to be used in one of the therapy sessions. All participants completed baseline body image measures (T1) after reaching a median BMI of 90% after which those in the treatment group commenced PBI treatment and those in the control group continued to receive TAU. After the first seven sessions of PBI, participants in the treatment group completed a second set of body image measures (T2) before commencing the ME part of the programme. All participants in both the treatment and control groups then completed a final set of body image measures (T3) on completion of the study at week 10. The body image measures completed at each time point were the EDE-Q, PASTAS, BIAQ and BIAAQ. For those in the treatment condition they also completed the feedback form at T3.

### Statistical analyses

This was a pilot study. The primary aim of the data analysis phase was not to definitively test whether PBI is effective or not but to generate evidence of its potential effectiveness, if progressed into a full trial. ANCOVA was used to estimate the difference in self-reported body image at week 10 between conditions, controlling for baseline self-reported body image as a covariate. The difference in mean self-reported body image scores is reported (adjusted for baseline levels) with 95% confidence intervals to indicate the potential effectiveness of the PBI intervention in comparison to TAU. The assumptions of normality and homogeneity of variance were assessed using the one-sample Kolmogorov–Smirnov test and the Levine’s test for the Equality of Error Variances.

The effect of ME was examined by estimating the mean difference in self-reported body image between week seven and week 10 in just the PBI group. This used paired data so the mean of the paired differences between weeks seven and 10 is presented with 95% confidence intervals.

In addition, to assess clinical significance, Cohen’s D effect sizes [38] are reported.

## Results

### Participant characteristics/descriptive statistics

Forty adolescent girls were recruited and randomised to the study. The mean age of the sample was 14.2 years (SD = 1.6) ranging from 11 to 17. Two of the 40 had a comorbid diagnosis of Autistic Spectrum Disorder (ASD). Mean age of onset of AN was 12.9 (SD = 1.6), ranging from 9 to 17. As part of TAU, 23 (57%) were receiving CBT-E, 15 (37%) were receiving psychodynamic psychotherapy, one had received both CBT-E and psychodynamic psychotherapy due to a change in therapy part way through treatment and one declined to attend individual therapy. Weight for height and BMI was collected for all participants at T1 and T3, and for PBI participants at T2 (see Table 2).

**Table 2:** Mean weight for height and mean BMI at T1, T2 and T3

Time point	PBI + TAU *(N *= *15)*				TAU *(N *= *16)*			
	Mean weight for height	SD	Mean BMI	SD	Mean weight for height	SD	Mean BMI	SD
1	91.84	1.41	17.91	0.87	91.96	3.96	17.95	0.86
2	98.08	2.62	19.19	0.82	–	–	–	–
3	97.87	2.26	19.19	0.89	98.5	4.29	19.33	0.97

Twenty participants were randomised to PBI and 20 to the control condition. Nine participants did not complete the study. Reasons for non-completion of the study included; team discharge prior to completion of research (*n *= 4), discharged against medical advice (*n *= 2), lost weight so no longer met the weight criterion for the research (*n *= 1) and withdrew consent from the research project (*n *= 2). Following discharge from inpatient services, participants are not followed-up for research purposes given the large changes in environmental factors between inpatient and community settings which would likely influence the results. Therefore, 31 participants completed the study and thus make up the dataset for this analysis; 15 in PBI and 16 in TAU.

Normality and homogeneity of variance were indicated, results not given here.

### Effectiveness of Practical Body Image intervention

Mean self-reported body image scores were calculated for the control and treatment group at the start (T1) and end of the programme (T3), see Table 3. It can be seen that mean scores decrease in both conditions between T1 and T3. However, the difference in mean scores at T3 (controlling for baseline outcome) indicate better self-reported body image in the treatment group in comparison to the control group for all outcomes. A positive difference in means indicates the PBI intervention having greater effect than control. Effect sizes range from 0.14 for Overall BIAAQ to 0.54 for BIAQ; Clothing. For six of the seven outcomes, the effect size was above 0.32.

**Table 3:** Comparison of conditions with respect to self-reported body image outcomes at week 10, mean difference (PBI + TAU vs TAU, controlling for baseline outcome), 95% confidence intervals (CI) and Cohens d

	TAU *(N *= *16)*				PBI + TAU *(N *= *15)*					
	T1		T3		T1		T3			
Measures	Mean	SD	Mean	SD	Mean	SD	Mean	SD	Mean difference (95% CI)	Cohens d
EDE-Q: Weight Concern	3.93	1.89	3.17	2.26	4.04	1.67	2.78	1.78	0.36 (− 0.74, 1.53)	0.50
EDE-Q: Shape Concern	4.61	1.80	3.98	2.10	4.75	1.40	3.47	1.65	0.49 (− 0.61, 1.59)	0.32
BIAAQ	60.69	20.38	53.00	22.50	57.79	16.41	50.13	15.92	2.87 (− 9.12, 14.85)	0.14
BIAQ: Clothing	2.38	0.88	2.24	0.98	2.92	1.02	1.73	0.83	0.50 (− 0.05, 1.05)	0.54
BIAQ: Social	3.05	1.24	2.35	1.60	3.20	1.14	1.86	1.05	0.49 (− 0.37, 1.36)	0.39
BIAQ: Grooming	3.13	0.78	2.92	0.73	3.03	1.05	2.59	1.08	0.33 (− 0.31, 0.96)	0.36
PASTAS: Traits	50.81	14.31	50.03	17.87	52.97	16.03	43.17	11.64	6.86 (− 1.42, 15.14)	0.45

*EDE*-*Q* Eating Disorders Examination Questionnaire*, BIAAQ* Body Image Acceptance and Action Questionnaire*, BIAQ* Body Image Avoidance Questionnaire, *PASTAS* Physical Appearance State Trait Anxiety Scale: Trait Version

### Changes in self-reported body image following ME intervention

Data were available for 14 of the 15 PBI participants who completed the study. Mean self-reported body image scores were calculated pre-ME (T2) and post-ME (T3), see Table 3. This analysis showed that at T3 all measures indicated better self-reported body image than at T2. For five of the seven measures the confidence intervals indicate statistically significant (*p* < 0.05) change in outcomes with within group effect sizes ranging from 0.27 to 0.43. The largest effect sizes, 0.42 and 0.43, were seen for body image avoidance in terms of clothing and grooming. The changes in shape concerns and body image avoidance in terms of social situations were not statistically significant (Table 4).

**Table 4:** Change in self-reported body image before and after ME, mean difference (95% CI) and Cohens d

Measures	T2 *(N *= *14)*		T3 *(N *= *14)*		Mean difference (95% CI)	Cohens d
	Mean	SD	Mean	SD		
EDE-Q: Weight Concern	3.53	1.84	3.03	2.84	0.50 (0.05, 0.95)	0.27
EDE-Q: Shape Concern	4.24	1.55	3.78	1.68	0.46 (− 0.03, 0.96)	0.30
BIAAQ	56.57	14.59	51.00	16.79	5.57 (1.59, 9.55)	0.38
BIAQ: Clothing	2.31	0.77	1.98	0.90	0.33 (0.06, 0.61)	0.43
BIAQ: Social	2.27	1.43	1.96	1.13	0.30 (− 0.09, 0.70)	0.21
BIAQ: Grooming	3.11	1.20	2.61	1.16	0.50 (0.22, 0.78)	0.42
PASTAS: Traits	49.14	11.50	46.07	12.00	3.07 (0.93, 5.21)	0.27

*EDE*-*Q* Eating Disorders Examination Questionnaire*, BIAAQ* Body Image Acceptance and Action Questionnaire*, BIAQ* Body Image Avoidance Questionnaire*, PASTAS* Physical Appearance State Trait Anxiety Scale: Trait Version

### Evaluation form

**Fig. 1:**
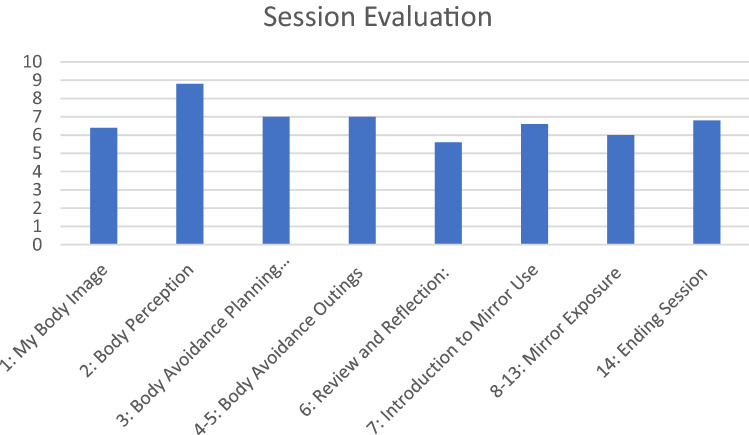
Helpfulness ratings of the participants following the intervention

The patients rated all sessions on a scale of 1–10 for how helpful they found them. Average helpfulness ratings have been calculated (see Fig. 1). Feedback showed the treatment was acceptable to users. The patients reported that the sessions were helpful in supporting body image concerns, “What I thought was helpful the most was probably getting evidence about my beliefs”. The sessions concerning errors of perception and negative beliefs were reported as most useful, “It helped me understand that how I perceived my body is different to what other people will think”. The ME was the least liked part of the programme, but rated as helpful in tackling anxiety, “I found ME really helpful and I can see the difference in how I feel about my body and shape” “I can now wear clothes that I couldn’t wear before”.

## Discussion

This study reports on the potential effectiveness and acceptability of PBI, when used as an adjuvant therapy in an inpatient unit. PBI is a new manualised individual therapy, specifically designed for children and adolescents with AN, to reduce body dissatisfaction. The study also investigates the therapeutic benefit of the ME component of PBI as an intervention for reducing the anxiety in relation to body dissatisfaction. Effectiveness was measured by self-report questionnaires and acceptability was assessed using qualitative data obtained from participants’ feedback.

Effect sizes suggest that PBI is beneficial for addressing body image dissatisfaction. Furthermore, the magnitude of the effect sizes on the EDE-Q (d = 0.32–0.50) are comparable to previous studies that have reported effect sizes on the same measure following body image intervention (d = 0.33–0.69) [17, 20, 21]. This suggests that the programme is of clinical importance and could be a promising new treatment for AN, certainly if offered in a setting that was not so therapeutically dense. Therefore, the programme may be more beneficial in an outpatient setting which typically offers fewer therapeutic interventions.

Furthermore, the qualitative feedback gained from the participants was positive and indicated that the intervention was acceptable to users. A number of individuals reported improvements related to body perception and beliefs about their body, in addition to an improvement in body avoidance behaviours such as feeling confident to wear clothes that they like.

These findings support previous research [16–23, 26] showing the effectiveness of CBT-based interventions for the treatment of body dissatisfaction. They also provide the first piece of empirical evidence to suggest that a CBT-based individual intervention is feasible for the treatment of body image dissatisfaction in children and adolescents with AN. Future research may consider comparing the effectiveness of PBI with group-based body image interventions as guidelines from the National Institute for Health Care and Excellence [39] recommend a comparison of group versus individual psychological interventions for eating disorders.

Following the ME element of the programme, there were statistically significant improvements in weight concerns, body image avoidance and trait anxiety in relation to physical appearance. This was also supported by small to medium effect sizes suggesting its clinical relevance. These findings add further support to previous research into the benefits of ME in treating body dissatisfaction [16, 27–29]. It should be noted that these findings are limited given data were not collected from the control group at T2; therefore, it is not possible to confirm that the ME intervention was solely responsible for the reduction in body image anxiety. Therefore, measured improvements in the treatment group may reflect a general trend towards recovery over time, or may be due to other elements of the inpatient treatment programme. All patients received additional psychological therapies (CBT-E or psychodynamic psychotherapy) throughout their admission which may have also contributed to changes in body dissatisfaction. Furthermore, therapeutic gains may be due to utilising skills and knowledge from the previous part of the PBI programme. Further controlled research is required to explore the use of ME in an adolescent population.

Research within the field of AN, and in inpatient settings in particular, is challenging. The patients’ ambivalence regarding recovery, clinical staff priorities and retention of participants due to variable admission lengths are all well-recognised problems [40].

The number of patients who were discharged prior to completion of the research highlights the difficulty of implementing therapeutic interventions in inpatient settings and raises the question of how to evaluate these effectively. Consideration of whether participants can remain in the study following discharge would enable retention of sample size but may likely confound the results given the stark differences between inpatient and community settings. The authors also recognise the inherent biases associated with investigating the effectiveness of an intervention that they were involved in developing and facilitating. Future research would benefit from independent researchers replicating these findings and including a follow-up measure to determine the stability of the effects found.

A strength of the PBI programme is the multi-faceted treatment approach to body image which targets the separate components of body image: cognitive, affective and behavioural. Further strengths include a TAU control group.

Body dissatisfaction is widely acknowledged as a core feature of AN, however, there is a limited evidence base regarding the effectiveness of an individual body image therapy for adolescents with AN. This study explored the effectiveness of a novel, manualised 14-session body image therapy with ME, to address negative body image and acceptance of a healthy weight. Overall, the findings suggest a specific body image programme does not detract from an intensive treatment programme which consists of many other active body image treatments. These results are particularly reassuring, because PBI was tested against TAU which included a battery of standardised treatments, including individual and group therapy, self-esteem therapy, exercise and activity groups, occupational therapy, family therapy, dietetic counselling, nursing support, leisure groups, schooling and medication prescribed by a Consultant Psychiatrist. CBT threads were in both the individual and group work.

These results suggest that the programme is of clinical importance and could be a promising new treatment for AN particularly when used as an adjuvant to other standard treatments. As well as adding to the existing literature, it is hoped that this study encourages further research into the use of CBT-based interventions for body image dissatisfaction in and particularly, the use of ME for adolescents with AN as the current literature is scarce.

## References

[1] American Psychiatric Association (2013) Diagnostic and statistical manual of mental disorders (DSM-5^®^). American Psychiatric Pub

[2] Bachner-Melman R, Zohar AH, Ebstein RP (2006). An examination of cognitive versus behavioural components of recovery from anorexia nervosa. J Nerv Ment Dis.

[3] Federici A, Kaplan AS (2008). The patient’s account of relapse and recovery in anorexia nervosa: a qualitative study. Eur Eating Dis Rev.

[4] Herpertz-Dahlmann B, Hebebrand J, Müller B, Herpertz S, Heussen N, Remschmidt H (2001). Prospective 10-year follow-up in adolescent anorexia nervosa—course, outcome, psychiatric comorbidity, and psychosocial adaptation. J Child Psychol Psychiatry Allied Discipl.

[5] Steinhausen HC (2002). The outcome of anorexia nervosa in the 20th century. Am J Psychiatry.

[6] Stice E (2002). Risk and maintenance factors for eating pathology: a meta-analytic review. Psychol Bull.

[7] Carter JC, Mercer-Lynn KB, Norwood SJ, Bewell-Weiss CV, Crosby RD, Woodside DB, Olmsted MP (2012). A prospective study of predictors of relapse in anorexia nervosa: implications for relapse prevention. Psychiatry Res.

[8] Keel PK, Dorer DJ, Franko DL, Jackson SC, Herzog DB (2005). Post-remission predictors of relapse in women with eating disorders. Am J Psychiatry.

[9] Rosewall JK, Gleaves DH, Latner JD (2018). An examination of risk factors that moderate the body dissatisfaction-eating pathology relationship among New Zealand adolescent girls. J Eat Disord.

[10] Farrell C, Shafran R, Lee M, Fairburn CG (2005). Testing a brief cognitive-behavioural intervention to improve extreme shape concern: a case series. Behav Cognit Psychother.

[11] Skrzypek S, Wehmeier PM, Remschmidt H (2001). Body image assessment using body size estimation in recent studies on anorexia nervosa. A brief review. Eur Child Adolesc Psychiatry.

[12] Mitchison D, Hay P, Griffiths S, Murray SB, Bentley C, Gratwick-Sarll K, Mond J (2017). Disentangling body image: the relative associations of overvaluation, dissatisfaction, and preoccupation with psychological distress and eating disorder behaviours in male and female adolescents. Int J Eat Disord.

[13] Tremblay L, Limbos M (2009). Body image disturbance and psychopathology in children: research evidence and implications for prevention and treatment. Curr Psychiatry Rev.

[14] Reas DL, Whisenhunt BL, Netemeyer R, Williamson DA (2002). Development of the body checking questionnaire: a self-report measure of body checking behaviours. Int J Eat Disord.

[15] Shafran R, Fairburn CG, Robinson P, Lask B (2004). Body checking and its avoidance in eating disorders. Int J Eat Disord.

[16] Morgan JF, Lazarova S, Schelhase M, Saeidi S (2014). Ten session body image therapy: efficacy of a manualised body image therapy. Eur Eating Disord Rev.

[17] Legenbauer T, Schütt-Strömel S, Hiller W, Vocks S (2011). Predictors of improved eating behaviour following body image therapy: a pilot study. Eur Eating Disord Rev.

[18] Bhatnagar KA, Wisniewski L, Solomon M, Heinberg L (2013). Effectiveness and feasibility of a cognitive-behavioural group intervention for body image disturbance in women with eating disorders. J Clin Psychol.

[19] Vocks S, Legenbauer T, Troje N, Schulte D (2006). Body image therapy in eating disorders. Influencing of perceptive, cognitive-affective, and behavioural components of the body image. Zeitschrift für klinische Psychologie und Psychotherapie.

[20] Mountford VA, Brown A, Bamford B, Saeidi S, Morgan JF, Lacey H (2015). BodyWise: evaluating a pilot body image group for patients with anorexia nervosa. Eur Eating Disord Rev.

[21] Rosewall JK, Beavan A, Houlihan C, Bates S, Melhuish L, Mountford V, Lacey JH (2019). Evaluation of Teen BodyWise: a pilot study of a body image group adapted for adolescent inpatients with anorexia nervosa. Eat Weight Disord Stud Anorexia Bulim Obes.

[22] Butters JW, Cash TF (1987). Cognitive-behavioural treatment of women’s body-image dissatisfaction. J Consult Clin Psychol.

[23] Grant JR, Cash TF (1995). Cognitive-behavioural body image therapy: comparative efficacy of group and modest-contact treatments. Behav Ther.

[24] Fairburn CG (2008). Cognitive behaviour therapy and eating disorders.

[25] Waller G, Cordery H, Corstorphine E, Hinrichsen H, Lawson R, Mountford V, Russell K (2007). Cognitive behavioural therapy for eating disorders: a comprehensive treatment guide.

[26] Calugi S, Dalle Grave R (2019). Body image concern and treatment outcomes in adolescents with anorexia nervosa. Int J Eat Disord.

[27] Key A, George CL, Beattie D, Stammers K, Lacey H, Waller G (2002). Body image treatment within an inpatient program for anorexia nervosa: the role of mirror exposure in the desensitization process. Int J Eat Disord.

[28] Moreno-Domínguez S, Rodríguez-Ruiz S, Fernández-Santaella MC, Jansen A, Tuschen-Caffier B (2012). Pure versus guided mirror exposure to reduce body dissatisfaction: a preliminary study with university women. Body Image.

[29] Delinsky SS, Wilson GT (2006). Mirror exposure for the treatment of body image disturbance. Int J Eat Disord.

[30] Butler RM, Heimberg RG (2020). Exposure therapy for eating disorders: a systematic review. Clin Psychol Rev.

[31] Ziser K, Mölbert SC, Stuber F, Giel KE, Zipfel S, Junne F (2018). Effectiveness of body image directed interventions in patients with anorexia nervosa: a systematic review. Int J Eat Disord.

[32] Fairburn CG, Beglin SJ (1994). Assessment of eating disorders: interview or self-report questionnaire?. Int J Eat Disord.

[33] Fairburn CG, Cooper Z, O’Connor M (1993). The eating disorder examination. Int J Eat Disord.

[34] Luce KH, Crowther JH (1999). The reliability of the eating disorder examination—self-report questionnaire version (EDE-Q). Int J Eat Disord.

[35] Reed DL, Thompson JK, Brannick MT, Sacco WP (1991). Development and validation of the physical appearance state and trait anxiety scale (PASTAS). J Anxiety Disord.

[36] Rosen JC, Srebnik D, Saltzberg E, Wendt S (1991). Development of a body image avoidance questionnaire. Psychol Assess.

[37] Sandoz EK, Wilson KG, Merwin RM, Kellum KK (2013). Assessment of body image flexibility: the body image-acceptance and action questionnaire. Journal of Contextual Behavioural Science.

[38] Cohen J (1992). A power primer. Psychol Bull.

[39] National Institute of Health and Care Excellence. (2017). Eating disorders: recognition and treatment. Retrieved from: www.nice.org.uk/guidance/NG69 28654225

[40] Bulik CM (2014). The challenges of treating anorexia nervosa. Lancet (London, England).

